# The influence of serotonin- and other genes on impulsive behavioral aggression and cognitive impulsivity in children with attention-deficit/hyperactivity disorder (ADHD): Findings from a family-based association test (FBAT) analysis

**DOI:** 10.1186/1744-9081-4-48

**Published:** 2008-10-20

**Authors:** Robert D Oades, Jessica Lasky-Su, Hanna Christiansen, Stephen V Faraone, Edmund JS Sonuga-Barke, Tobias Banaschewski, Wai Chen, Richard JL Anney, Jan K Buitelaar, Richard P Ebstein, Barbara Franke, Michael Gill, Ana Miranda, Herbert Roeyers, Aribert Rothenberger, Joseph A Sergeant, Hans-Christoph Steinhausen, Eric A Taylor, Margaret Thompson, Philip Asherson

**Affiliations:** 1 Clinic for Child and Adolescent Psychiatry and Psychotherapy, University of Duisburg-Essen, Essen, Germany; 2 Channing Laboratory, Brigham and Women's Hospital, Boston, MA, USA; 3 Departments of Psychiatry, Neuroscience and Physiology, SUNY Upstate Medical University, Syracuse, NY, USA; 4 Developmental Brain-Behavior Unit, School of Psychology, University of Southampton, Southampton, UK; 5 Department of Child and Adolescent Psychiatry and Psychotherapy, Central Institute of Mental Health, J 5, Mannheim, Germany; 6 Developmental Brain-Behavior Unit, School of Psychology, University of Southampton, Southampton, UK; 7 Department of Psychiatry, Trinity Centre for Health Sciences, St. James's Hospital, Dublin, Ireland; 8 Department of Psychiatry, Radboud University Nijmegen Medical Center, Nijmegen, The Netherlands; 9 S. Herzog Memorial Hospital, Jerusalem, Israel; 10 Department of Human Genetics, Radboud University Nijmegen Medical Center, Nijmegen, The Netherlands; 11 Department of Developmental & Educational Psychology, University of Valencia, Spain; 12 Department of Experimental Clinical and Health Psychology, Ghent University, Belgium; 13 Department of Child and Adolescent Psychiatry, University of Göttingen, Germany; 14 Vrije Universiteit, De Boelelaan 1105 1081 HV, Amsterdam, The Netherlands; 15 Department of Child & Adolescent Psychiatry, University of Zurich, Switzerland; 16 MRC Social Genetic Developmental & Psychiatry Centre, Institute of Psychiatry, London, UK; 17 Child Study Center, New York University, New York, USA

## Abstract

**Background:**

Low serotonergic (5-HT) activity correlates with increased impulsive-aggressive behavior, while the opposite association may apply to cognitive impulsiveness. Both types of impulsivity are associated with attention-deficit/hyperactivity disorder (ADHD), and genes of functional significance for the 5-HT system are implicated in this disorder. Here we demonstrate the separation of aggressive and cognitive components of impulsivity from symptom ratings and test their association with 5-HT and functionally related genes using a family-based association test (FBAT-PC).

**Methods:**

Our sample consisted of 1180 offspring from 607 families from the International Multicenter ADHD Genetics (IMAGE) study. Impulsive symptoms were assessed using the long forms of the Conners and the Strengths and Difficulties parent and teacher questionnaires. Factor analysis showed that the symptoms aggregated into parent- and teacher-rated behavioral and cognitive impulsivity. We then selected 582 single nucleotide polymorphisms (SNPs) from 14 genes directly or indirectly related to 5-HT function. Associations between these SNPs and the behavioral/cognitive groupings of impulsive symptoms were evaluated using the FBAT-PC approach.

**Results:**

In the FBAT-PC analysis for cognitive impulsivity 2 SNPs from the gene encoding phenylethanolamine N-methyltransferase (PNMT, the rate-limiting enzyme for adrenalin synthesis) attained corrected gene-wide significance. Nominal significance was shown for 12 SNPs from BDNF, DRD1, HTR1E, HTR2A, HTR3B, DAT1/SLC6A3, and TPH2 genes replicating reported associations with ADHD. For overt aggressive impulsivity nominal significance was shown for 6 SNPs from BDNF, DRD4, HTR1E, PNMT, and TPH2 genes that have also been reported to be associated with ADHD. Associations for cognitive impulsivity with a SERT/SLC6A4 variant (STin2: 12 repeats) and aggressive behavioral impulsivity with a DRD4 variant (exon 3: 3 repeats) are also described.

**Discussion:**

A genetic influence on monoaminergic involvement in impulsivity shown by children with ADHD was found. There were trends for separate and overlapping influences on impulsive-aggressive behavior and cognitive impulsivity, where an association with PNMT (and arousal mechanisms affected by its activity) was more clearly involved in the latter. Serotonergic and dopaminergic mechanisms were implicated in both forms of impulsivity with a wider range of serotonergic mechanisms (each with a small effect) potentially influencing cognitive impulsivity. These preliminary results should be followed up with an examination of environmental influences and associations with performance on tests of impulsivity in the laboratory.

## Background

Impulsive actions usually damage not only the victim but often their perpetrator. In fights, suicide or fire-setting the aggressive component is immediately apparent. But the risky and overly rapid decisions behind these actions demonstrate a cognitive component that is often revealed in gambling and seeking immediate gratification. Impulsivity is a core construct of many mental disorders [1]. In particular, it is an essential component of the diagnosis of the most prevalent form of attention-deficit/hyperactivity disorder ADHD, namely the 'combined type' (ADHD-ct: [2]).

Across a group of youngsters with ADHD-ct there are many who show abrupt bursts of impulsive aggression (related to conduct disorder), but many others who (merely) make rapid and wrong decisions in everyday life, or abhor delayed gratification to their cost [3]. Here, we consider that these two forms of impulsivity may have distinct pathophysiological features with relation to ADHD. In particular, this article focuses on serotonin activity, [4]) and heritable features of ADHD, where a 70–80% heritability has been clearly demonstrated [5].

Impulsivity has been briefly and broadly defined as "action without foresight" [6]. More generally impulsivity covers actions that appear "poorly conceived, prematurely expressed, and are unduly risky or inappropriate to the situation. They often result in undesirable consequences" [7]. Impulsivity has been linked strongly to outbreaks of aggression in adults (here considered as overt impulsive behavioral aggression) that are associated with increased 5-HT2a platelet receptor binding (Bmax and Kd), decreased 5-HT1a postmortem binding and low serotonin (5-HT) activity [8-10]. While some studies have failed to find the same relationship of low 5-HT activity with aggression in children [11-13] many others do report an inverse relationship [14-16] sensitive to pharmacotherapy [17]. It may be that the phenocopy for aggression has not always fully developed at the time of study. Impulsive aggression reacts to provocation and is marked after 5-HT synthesis reduction following tryptophan depletion [18]. Indeed, Halperin's group, in showing an apparently protective effect of high 5-HT activity, recently proposed that low activity is important if not sufficient for the emergence of aggression. Consistent with the developmental expression of the trait they reported that low 5-HT responsivity in childhood predicted the development of antisocial personality disorder 9 years later [19].

More differentiated views of impulsiveness [20] suggest that, as well as aggressive and behavioral impulsiveness associated with emotional lability and motor control, there are also cognitive or attention-related elements [21]; for example, there are the cognitive expressions of impulsiveness that are associated with failures of inhibitory executive functions ("executive attention", [22]). Typically decisions are made hastily and lead to maladaptive outcomes. Such impulsivity is epitomized by errors of commission on a discrimination or Go/no-go task, and is perhaps reflected in more motivationally-based domains where response choice is driven by early reinforcement [23]. This form of cognitive impulsiveness may be regarded as a form of impaired selective attention. However, it is a result of overly rapid, inadequate information processing that leads to inappropriate action. This should be contrasted with absences of conventional attentive processes (inattentiveness) that would normally bring the searchlight, the focus of selective processing to stimulus identification. Inattention leads to the omission of a response. Thus it is important to distinguish the term inattention, as it is usually understood, from impulsive (cognitive) processing that risks non-adaptive responses. Such impulsive decisions about the moment-to-moment organization and planning of responses to stimuli are commonly made by both younger [24] and older subjects with ADHD [25].

The goal of the current study is to disentangle the relationship between aggressive impulsivity of a more behavioral nature and cognitive impulsivity in ADHD by exploring the differential patterns of association of these two forms of impulsivity with genes that have significance for 5-HT function, a putative neurobiological marker of impulsivity. There is mounting evidence that polymorphisms for genes influencing several features of the 5-HT system are associated with ADHD [4], and a case has been made for a differential involvement of 5-HT activity in impulsive aggressive behavior and cognitive impulsivity [26]. For example, in one study increases of platelet 5-HT transporter affinity in 20 children with ADHD correlated with ratings of externalizing behavior, but decreases of affinity were associated with distractibility, impulsivity and the difficulty to withhold a prepotent response on the stop-task [27]. Similarly a study of adolescent prisoners described opposite correlations of 5-HT activity with crime and other measures of impulsivity [28]. On tasks assessing impulsivity (see [29]), errors of commission are typical of youngsters with ADHD who show an underactivation in the right frontal cortices [30]. An impairment is expressed in similar regions of young healthy subjects treated with a tryptophan-depleting drink [31].

We therefore explored whether there would be commonalities and differences between the two forms of impulsivity and 5-HT genes controlling the activation or suppression of activity within 5-HT systems, along with other genetic markers of neuronal activity (e.g. dopamine) which influence or are influenced by the activity in 5-HT systems [32].

## Methods

### Participants

The initial data-set contained 614 families with 776 European Caucasian cases of DSM-IV ADHD-ct and 706 siblings without the disorder. These families had been recruited as part of the International Multicenter ADHD Genetics Project (IMAGE: details in [33]). They had been included in the first stage of genotyping, which investigated 51 candidate genes for the disorder [24]. In this sample, 1180 offspring from 607 families had the necessary genotype and phenotype data to be included in the analysis. These individuals were recruited from 12 specialist clinics in 8 European countries (Belgium, Germany, Ireland, Israel, Spain, Switzerland, the Netherlands and the United Kingdom). The sample was aged 5–17 years on entry to the study (mean ages for both groups were 10.9 y: table 1). In 4 families the case did not meet the strict criteria for ADHD-ct. However, these families were included in the study because they either 1) met the criteria for inattentive or hyperactive-impulsive ADHD or 2) were just below the threshold for meeting the ADHD-ct criteria. As genetic association studies are underpowered, we felt that including these individuals in the analysis would be beneficial by keeping the sample size higher.

**Table 1:** Sample Characteristics

Number of genotyped families	607
Total number of genotyped people	2337

Number of genotyped parents	1157

Number of genotyped offspring	1180

Number of offspring per family	
1	124
2	402
3	72
4	9

Gender of offspring:	
Male	793 (67.2%)
Female	387 (32.8%)

Average age of offspring (Standard Deviation)	10.9 (3.0)

Number of affected offspring	603

Origin:	
Northern Europe	882
Spain	99
Israel	199

Note: Diagnosis was restricted to children (ADHD combined type) and not systematically attempted with parents.

Exclusion criteria were genetic or medical disorders associated with behavior mimicking ADHD, autism, epilepsy, IQ<70, and the absence of a medication-free period permitting evaluation in the previous 2 years. Approval for the study was obtained from ethical review boards at each clinic, which were registered with and monitored by the National Institutes of Health, and were in accordance with the Code of Ethics of the World Medical Association (Declaration of Helsinki).

### Assessments: diagnosis and impulsivity

Clinical assessments were made during medication free periods or based on recall of such periods in the last 2 years. Diagnoses of ADHD-ct were based on the semi-structured Parental Account of Children's Symptoms (PACS; [34]). Interviewers received formal training in administration of the PACS. PACS includes 4 subscales, representing the different DSM-IV categories for ADHD and disruptive behaviors (hyperactivity, defiance, emotionality, and comorbid disorders). An age adjustment for symptom thresholds is built into the PACS algorithm. Detailed descriptions of the child's typical behavior in a range of specified situations defined by the context (e.g. play) or the behavior shown (e.g. crying) that had occurred in the previous week and in the previous year were rated on a 4-point scale for frequency and severity. Situational pervasiveness was determined by the different situations investigated within the interview and the presence of at least one symptom in each domain reported in the Conners teacher rating (CTRS) ADHD sub-scales. Autism spectrum disorders were screened for all children using the Social Communication Questionnaire (≥ 15) in conjunction with the pro-social scale from the Strengths and Difficulties Questionnaire (SDQ ≤ 4). Inter-rater reliability was high with product-moment correlations for pairs of interviewers ranging from 0.79 to 0.96. A mean kappa coefficient across all the sites of 0.88 (range 0.71–1.00) and an average agreement percentage of 96.6% (range 78.6–100) were obtained. Concurrent validity of the PACS diagnosis was confirmed by the biserial correlation between PACS diagnosis of ADHD-ct with the CTRS N-scale (18 DSM-items) scores at 0.68 and with Conners' Parent rating scale (CPRS) N-scale scores at 0.78 [34-36].

For both the cases and the siblings, parents completed the long form of the revised CPRS (80 items) and teachers the long form of the revised CTRS (59 items [37]). Both also completed the SDQ (25 items). The SDQ scores are based on 5 different scales: emotional problems, behavior problems, hyperactivity/inattention, peer problems, and prosocial behavior. The scale is well validated and has good test-retest reliability (α = .85: [38,39]). From these parent and teacher rating scales (CPRS, CTRS, SDQ) we selected symptoms with impulsive behavioral aggression and cognitive impulsiveness. The 47 items selected are listed in tables 2 and 3 where the source of each item is indicated by the code in the legend.

**Table 2:** Principle components analysis (oblique rotation) of 47 items of impulsivity: Parent and teacher ratings from Conners and SDQ questionnaires (CP, SP and CT, ST, respectively).

Part 1 – The aggressive behavioral form of impulsivity (factors 1 and 2)			
**Item**	**Descriptor**	**Factor 1**	**Factor 2**

CP47	Temper outbursts	0.950	

CP21	Loses temper	0.893	

SP5	Often has temper tantrums	0.896	

CP31	Irritable	0.840	

CP57	Touchy or easily annoyed	0.859	

CP77	Mood changes quickly	0.820	

CP1	Angry and resentful	0.916	

CP40	Actively defies or refuses to	0.780	

CP11	Argues with adults	0.826	

CP68	Demands to be met at once	0.598	

CP28	Excitable, impulsive	0.356	

CP70	Spiteful or vindictive	1.000	

SP12	Often fights with other children	0.588	

CP66	Disturbs other children	0.294	

CP64	Get upset if someone rearranges	0.947	

SP7	Generally obedient, usually ...	-0.510	

ST5	Often has temper tantrums		1.000

CT7	Temper outbursts; explosive,		0.972

CT45	Demands must be met at once		0.898

CT54	Mood changes quickly, drastically		0.945

CT37	Argues with adults		0.957

CT55	Interrupts or intrudes on others		0.655

CT8	Excitable, impulsive		0.661

CT29	Has difficulty waiting in turn		0.606

CT46	Blurts out answers to question		0.674

ST12	Often fights with other children		0.922

CT47	Spiteful or vindictive		0.989

CT20	Leaves seat in classroom or		0.544

ST7	Generally obedient, usually		-0.700

ST25	Good attention span sees		-0.958

Variance Explained		14.6	13.2

Eigen Value		20.4	4.42

Squared Multiple Correlation		0.92	0.90

**Table 3:** Principle components analysis (oblique rotation) of 47 items of impulsivity: Parent and teacher ratings from Conners and SDQ questionnaires (CP, SP and CT, ST, respectively).

Part 2 -The cognitive form of impulsivity (factors 3 and 4)			
**Item**	**Descriptor**	**Factor 3**	**Factor 4**

CP79	Easily distracted by extraneous ...	0.886	

CP37	Fails to finish things started	1.000	

SP15	Easily distracted, poor concentration	0.924	

CP12	Fails to complete assignment	0.938	

CP48	Get distracted even when instructed	0.827	

CP29	Does not follow instructions through	0.872	

CP80	Blurts out answers to questions	0.694	

CP42	Has difficulty waiting in line	0.601	

SP21	Thinks things out before acting	-0.818	

SP25	Good attention span, sees ...	-0.961	

CT26	Inattentive easily distracted		0.865

CT52	Distractibility, or attention span poor		0.858

ST15	Easily distracted, poor concentration		0.842

CT58	Easily distracted by extraneous		0.713

CT57	Does not follow instructions through		1.000

CT17	Fails to finish things started		0.922

ST21	Thinks things out before acting		-0.481

ST25	Good attention span sees		-0.958

Variance Explained		16.0	12.8

Eigen Value		2.53	1.07

Squared Multiple Correlation		0.87	0.88

Item-codes from scales include C, Conners; S, Strengths and difficulties; P, Parent; T, Teacher. The combination of factors 1 and 2 relate to the behavioral aggression component, and factors 3 and 4 relate to the cognitive impulsivity component. Note: If represented as the sum, each form of impulsivity explained 28–29% of the variance. As the informants' ratings correlated highly the true contribution of the components would be approximately halved.

### Gene selection

We selected 14 genes with 582 single nucleotide polymorphisms (SNPs) from the data available from the IMAGE study for the core analysis reported here. For the 5-HT system, markers for pre- and post-synaptic receptors (HTR1A, HTR1B, HTR1E, HTR2A, HTR3B), and synaptic availability controlled by synthesis (TPH2) and re-uptake (SERT/SLC6A4) were chosen. We reasoned that some dopaminergic (DA) biomarkers should be included in view of widespread interactions between 5-HT and DA systems in the brain (reviews in [32,40]). These included SNPs for the receptors DRD1, DRD4 and DAT1/SLC6A3. There have been reports of associations for each of these genes with ADHD that were subsequently replicated (reviews: [4,5,41]). We also decided to include 4 genes for comparison that might be expected to have a less specific outcome on neural activity and behavior than those targeting neurotransmitter synthesis and receptor function (BDNF, NURR-1, FADS2, and PNMT: see discussion). For an additional analysis genotype information on repeat polymorphisms (microsatellites) was available for the DRD4 (exon 3 variable number tandem repeat [VNTR]), the DAT1/SLC6A3 (3' untranslated region VNTR), the SERT/SLC6A4 (a VNTR polymorphism in intron 2: STin2) and an in/del promoter polymorphism (5-HTTLPR), from an analysis based on 1116 ADHD families.

### DNA and SNP collection

The DNA sample used here is a part of a larger genetic sample from the IMAGE study. Details of the DNA isolation and SNP selection can be found elsewhere [33]. Briefly, blood samples were sent to Rutgers University Cell and DNA repository, New Jersey, U.S.A., where DNA was extracted. In a limited number of cases where individuals were not able to supply a blood sample, DNA was extracted from a mouth swab sample at the SGDP laboratories in London, U.K. [42]. DNA stocks were collated in London where they were stored, organized and plated out for further analysis. Geneservice Ltd. Cambridge (UK) performed whole genome amplification on all samples with less than 100 μg stock DNA, using the REPLI-g kit (Quiagen Ltd., Crawley, UK). DNA samples were arrayed into 96-well plates at a concentration of 50 ng/μl and delivered to Illumina Inc. (San Diego, US) under dry ice. A total of 582 SNPs were genotyped throughout 14 genes, as part of a larger set of SNPs/genes described elsewhere [33]. These SNPs were selected as tag-SNPs to explain the linkage disequilibrium structure throughout the gene. Additional polymorphisms genotyped were the repeat polymorphisms in DRD4, SLC6A3/DAT1 and SLC6A4/SERT. Genotyping was by simple sequence analysis on agarose gel, as described previously [43-45]. Each polymorphism was evaluated to ensure Hardy-Weinberg equilibrium, initially at α = 0.0001, then at α = 0.05. Data cleaning procedures were performed previously on the entire IMAGE set of SNP data and have been described [33].

### Analytical strategy: family based association test – principle components (FBAT-PC)

To determine that the 47 symptoms selected for association analysis would indeed group into major factors of cognitive and aggressive behavioral impulsivity we ran a preliminary factor analysis (Promax) with an oblique rotation in the SAS statistical package. This rotation is in line with clinical experience that subjects present concurrently with symptoms from different dimensions [46]. Four factors based on an eigenvalue cutoff of = 1.0 confirmed these groupings: namely, A) impulsive aggressive behavior based on 1) parent ratings, 2) teacher ratings, and B) cognitive impulsivity rated by 3) parents, and 4) teachers (tables 2 and 3). The names of these groupings reflect the items that emphasize temper outbursts and anger (2A), and distraction (despite understanding the task) that reflects responding to inappropriate stimuli (commission rather than omission: 2B). A look at tables 2 and 3 shows that few variables loading on one factor had strong weightings on the other. Three examples for factors 1 and 2 are "excitable impulsive" (CP28), "disturbs other children" (CP66) and "generally obedient" (SP7). Of interest is the item "blurts out answers before the question is finished" that loads modestly on factor 2 (CT46) with a similar value to that on factor 3 (CP80). This item could be construed as an example of impulsive behavior overlying a cognitively impulsive decision. A previous factor analysis of ADHD symptom domains in 10–14 y-old children confirmed that behavioral impulsivity related to aggression and delinquency, while inattentive items related to hyperactivity [47].

We then performed separate association analyses at each SNP for each of the two groupings while applying the FBAT-PC approach. The FBAT-PC approach generates a composite phenotype by using a weighted sum of the individual phenotypes (details in [48,49], for applications see [50,51]). The weights for this composite phenotype are selected to maximize the heritability at each SNP.

Screening procedures were employed to limit the number of statistical tests performed using the PBAT computer program. An outline of the screening procedure can be summarized as follows: 1) the conditional mean model is used to calculate the power to detect an association using the composite phenotype; 2) SNP/genetic model combinations are selected based on the power to detect an association and then ranked; 3) for the top ten power rankings in the FBAT-PC statistics are calculated for the selected SNP/genetic model combinations and their associated phenotypes; 4) the results are adjusted for multiple comparisons using the False Discovery Rate (FDR: [52]. For these analyses, we considered additive (0), dominant (1) and recessive (2) genetic models. We required our results to have at least 20 informative families.

## Results

### Sample description

Table 1 summarizes the characteristics of the IMAGE sample used in this analysis. There were 607 families, each with between 1 and 4 offspring who had complete genotype and phenotype information. Two thirds of the offspring were male and 603 had a diagnosis of ADHD-ct. The predominant ethnic make-up of the sample was northern European with subsets from Spain and Israel. As family-based tests are robust to population stratification all individuals were included in the analysis. Previous analyses of these data have shown that the findings did not change substantially when the Spanish and Israeli groups were removed.

### FBAT-PC analysis

We applied the FBAT-PC approach to the items making up 'impulsive aggression' and 'cognitive impulsivity' variables separately. For both analyses the associations were ranked by power and the first ten were selected for further consideration. No SNPs survived gene-wide corrections for an association with both forms of impulsivity.

For impulsive behavioral aggression there were 6 SNPs from 5 genes that attained nominal significance (p ≤ 0.05: table 4). These genes were BDNF, DRD4, HTR1E, TPH2, and PNMT.

**Table 4:** FBAT-PC results for behavioral (factors 1 + 2) and cognitive impulsivity (factors 3 + 4)

**Factors 1 & 2 (behavioral impulsivity)**									
Gene	SNP	Allele	Frequency	Model	Under/Over Transmitted	Inform. Families	p-value	Power	FDR p-value

*Nominal significance*									

BDNF	rs7103411	C	0.20927	2	O	91	0.021263	9	0.10746

BDNF	rs10767664	T	0.20889	2	O	87	0.021492	10	0.10746

DRD4	rs3758653	C	0.17287	2	O	52	0.008178	4	0.08178

HTR1E	rs1406946	A	0.44805	1	U	338	0.031630	1	0.26692

PNMT	rs2934966	T	0.04997	0	O	102	0.027007	2	0.10349

TPH2	rs6582071	A	0.22517	2	U	95	0.010714	10	0.10714

**Factors 3 & 4 (cognitive impulsivity)**									

**Gene wide significance**									

PNMT	rs200173	A	0.02383	0	O	40	0.003818	1	0.00936

PNMT	rs200173	A	0.02383	1	O	40	0.004680	4	0.00936

*Nominal significance*									

BDNF	rs13306221	A	0.05762	1	O	140	0.042211	6	0.22758

DRD1	rs686	G	0.35975	0	U	427	0.006978	6	0.06978

DRD1	rs686	G	0.35975	1	U	336	0.023762	8	0.11881

HTR1E	rs1406946	A	0.44805	1	O	338	0.034898	1	0.38387

HTR2A	rs6561333	T	0.43307	2	U	288	0.012237	4	0.12237

HTR2A	rs1923886	C	0.41760	2	U	278	0.033097	5	0.16548

HTR3B	rs1672717	C	0.39106	2	U	234	0.040249	4	0.20125

HTR3B	rs3782025	C	0.45063	2	U	266	0.026497	6	0.20125

SLC6A3	rs13189021	T	0.23494	2	U	103	0.018997	9	0.18997

TPH2	rs1352250	A	0.43298	1	O	359	0.011413	2	0.06598

TPH2	rs10879352	T	0.39596	1	O	364	0.015048	4	0.06598

TPH2	rs1487275	G	0.28429	1	O	356	0.019794	9	0.06598

Model: 0 = additive, 1 = dominant, 2 = recessive

Under/Over Transmitted: O = over-transmitted, U = under-transmitted

For cognitive impulsivity one SNP from the PNMT gene attained gene-wide statistical significance following an FDR correction for multiple testing in both additive and dominant models (p ≤ 0.05: table 4). In addition, 12 SNPs from 7 genes attained nominal significance (p ≤ 0.05) for an association with cognitive impulsivity. These genes were BDNF, DAT1/SLC6A3, DRD1, HTR1E, HTR2A, HTR3B, and TPH2.

Genotype information on repeat polymorphisms (microsatellites) was available for the DRD4 (exon 3, VNTR), the DAT1/SLC6A3 (3' untranslated region VNTR) and the SERT/SLC6A4 (a VNTR in intron 2: STin2) and an in/del promoter polymorphism (5-HTTLPR) from an analysis based on 1116 ADHD families. There were nominally significant associations on an additive model for the 3-repeat DRD4 allele (p = 0.014) with impulsive aggressive behavior, and the SERT VNTR (STin2: 12 *vs*. 9 and 10 repeat alleles; p = 0.050) with cognitive impulsivity. Other tests for association (e.g. for the promoter region, 5-HTTLPR long and short repeats) proved not to be significant.

Having identified the SNP/model combinations listed in table 4, we examined the associations with ADHD affection status. In addition to being the only gene where there was gene-wide significance, PNMT was also the only gene that was nominally associated with ADHD affection status. SNP rs200173 had associations with ADHD affection status for both the additive and dominant genetic models (unadjusted p-value = 0.0065 and 0.012 respectively) and rs2934966 had an association with the dominant model (unadjusted p-value = 0.0458).

## Discussion

After separating the 47 items from the SDQ and Conners symptom ratings into the two dimensions of aggressive and cognitive impulsivity, we used the FBAT-PC software to evaluate their possible associations with 582 SNPs distributed through 14 relevant genes. The genes were selected because their expression affects 5-HT activity and closely-related neural functions and they have previously been reported to be associated with a diagnosis of ADHD. The focus on 5-HT-related features was based on descriptions of the relevance of this transmitter to the expression of impulsivity [4,26,53] and for the preferential transmission of polymorphisms influencing 5-HT function in ADHD (see introduction). Gene-wide significance was achieved only for a variant of the gene encoding the enzyme for the synthesis of adrenalin (PNMT) in cognitive impulsivity. However nominal significance was shown for genes affecting a range of elements of the 5-HT and DA systems in both forms of impulsivity, and that have previously been reported to be associated with ADHD.

The absence of prominent associations for neurotransmitter markers, apart from that with adrenalin synthesis through PNMT variants, was surprising. A gene-wide significant association was found for one SNP for PNMT with cognitive impulsivity, and a second was nominally associated with behavioral aggressive impulsivity from the 4 SNPs for PNMT that were examined. In both instances the trait was over-expressed (table 4). This was partly anticipated by a similar significant finding in a candidate gene analysis for association with ADHD from the IMAGE sample [33]. PNMT is the rate-limiting enzyme in the synthesis of adrenalin that has transmitter and endocrine influences on brain function which can be stimulatory or inhibitory, respectively [54]. The association of adrenalin with CNS arousal is significant in the context of the common attribution of hypoarousal to ADHD function (review: [55]). Further, one might anticipate that arousal levels would be pertinent to both forms of impulsivity under investigation here, but especially the cognitive attention-related form that could be a precursor to the behavioral, aggression-related form. Indeed, adrenalin levels in ADHD children are usually lower than normal [56-58] unless they show anxiety [59-61]. Low levels are associated with restlessness and aggressive outbursts [62]. Response to treatment with methylphenidate tends to normalize levels of adrenalin [58,63] and improve autonomic measures of low arousal (skin conductance levels: [64]). Thus there is broad support for a widespread role for impaired function of arousal and adrenalin systems in ADHD and the impulsivity that is characteristic of the disorder. From our current results, we would predict that this could be based in an altered expression or function of the PNMT gene. Considering the potential parallel involvement of 5-HT systems in impulsivity, and the demonstrated role of 5-HT in the control of autonomic function [65] it would be useful to look for tagging between variants influencing PNMT and 5-HT receptors.

In agreement with our expectations, analyses of the two forms of impulsivity are reflected at least nominally in similar but also in different associations with the genetic markers for monoamine activity. For the 5-HT system, association with synthesis (TPH2) and receptor-mediated mechanisms (HTR1E) may be relevant to both forms of impulsivity. Indeed the same SNP for HTR1E (rs1406946) was nominally associated with both types of impulsiveness in the dominant transmission model. It was over-expressed in the cognitive and under-expressed in the behavioral or aggressive form of impulsivity (table 4). The difference for 5-HT function is one of emphasis, with a number of markers pointing to small effects on synthesis (TPH2 3 SNPs), pre-and post-synaptic receptors (HTR1E, HTR2A, HTR3B: 5 SNPs) and reuptake (SERT: STin2) for cognitive (not behavioral) impulsivity. For cognitive impulsivity 4 SNPs for receptors (apart from HTR1E) were under-expressed in a recessive model, whereas the availability of 5-HT (e.g. TPH2 in a dominant model) was influenced by over-expression of relevant alleles. For the DA system, nominally significant markers were found for both types of impulsivity, but while the emphasis for the behavioral/aggressive form was on DRD4 (one SNP and in particular the 3-repeat allele was over-expressed), cognitive impulsivity was nominally associated with uptake (DAT1: 1 SNP) and the D1 receptor (DRD1: 2 SNPs), which were all under-expressed. Based on their anatomical distributions, this could reflect top-down mesocortical and bottom-up mesolimbic mechanisms of control, respectively [66].

The 5-HT component implies a potential influence of 5-HT activity in those children with ADHD who show aggressive behavioral and cognitive impulsivity both through its availability (TPH2-mediated synthesis and SERT-mediated uptake), and the efficacy of 5-HT action via the 5-HT2 and 5-HT1 receptor families [67]. Generalizing in this context, activation of the 5-HT1B and 5-HT2A receptors tend to be 'stimulatory', while the 5-HT1A and 5-HT2C sites are more often 'inhibitory' [68]. The 5-HT receptor mediated influence obtains especial significance with regard to the interaction of 5-HT with the DA system considering that ADHD is often considered a 'hypodopaminergic' disorder [69,70] and the 5-HT and DA systems interact directly and widely through subcortical and cortical regions [32]. Even if the genetic effects reported here are modest, there is intriguing support for our hypothesis of opposing types of interactions of DA and 5-HT in the two forms of impulsivity. Thus, in aggressive behavioral impulsivity a DRD4 SNP was overexpressed while those for HTR1E and TPH were underexpressed. In contrast in cognitive impulsivity SNPs for DRD1 and DAT1 were underexpressed while others for TPH2 and HTR1E were overexpressed.

Corrected significant associations for the 5-HT2A gene have also been reported recently for children and adults with ADHD [71] and correlations with hyperactive/impulsive symptoms were described for a normal population [72]. However, other groups have seen no association [73,74]. Unfortunately, little is known about the functional role of 5-HT1E sites that make up a quarter of those binding 5-HT in the forebrain in autoradiographic postmortem studies of cortical and hippocampal regions (CA3-4 >> CA1 [75]). In contrast, 5-HT3 receptors have been more widely studied, and are abundant in limbic and frontal regions [76]. In contrast to the inhibitory functions attributed to HT1 receptors, stimulation of HT3 sites enhances DA release [77]. Thus the nominal significance of the association of 4 SNPs for stimulatory receptors (HT2A and HT3B) with cognitive impulsivity is consistent with the opposing effects of 5-HT activity on the two forms of impulsiveness proposed in the introduction.

The presence of 3 SNPs for TPH2 with near gene-wide significant associations with cognitive impulsiveness along with the significant relationship for a SERT allele (as also indicated by [78]) would suggest that markers for the presynaptic availability of 5-HT merit continued attention. The importance of this is illustrated, for example, by the functional consequence of changes in SERT activity as expressed with the short allele. In this instance the binding potential of 5-HT receptors may alter, as demonstrated for the 5-HT1a binding site [79]. In turn changes at this binding site (with inhibitory function) would strongly modulate transmitter release in DA systems. Considering the well-known interactions of these two monoaminergic systems [32], the potential for tagging between genetic loci should be sought in future studies.

Lastly, we studied SNPs representing 3 other genes (BDNF, FADS2, NURR1/NR4A2) on the grounds that their activity could influence neurotransmission including that based on 5-HT, and that some associations with ADHD have been reported [33]. Brain derived neurotrophic factor (BDNF) facilitates the development of the release mechanisms for several neurotransmitters. It is known to have age-dependent effects on 5-HT transporter function [80]. In BDNF-knockout mice this led to reduced 5-HT metabolism [81] and aggressive impulsive behavior [82]. A nominal association with both forms of impulsiveness emerged from the present analysis, that may reflect a small effect on the development of monoamine systems in general for cases of ADHD that particularly reflect an etiology of delayed maturation [83]. However, there is not much support for an association of BDNF markers with the diagnosis of ADHD [84]. NURR1/NR4A2 is an orphan nuclear receptor and transcription factor crucial to the genetic control of development of DA neurons [85,86]. The expression of NURR1/NR4A2 is essential for the induction of several markers of DA activity (e.g. tyrosine hydroxylase). However, there was no evidence here for the altered expression or function of NURR1/NR4A2. Finally, fatty-acid desaturase gene-2 (FADS2) is involved in regulating membrane synthesis and function and at least one polymorphism has been closely associated with ADHD [87]. The ratio of omega-3 to omega-6 unsaturated fatty acids in neural membranes certainly influences their fluidity and the ability of neurons to conduct efficiently: this is a problem for ADHD with documented white matter anomalies [88] and potentially for the function of oligodendrocytes responsible for membrane synthesis [89]. But we found no evidence that the FADS2 markers investigated were associated with the types of impulsivity studied here.

### Limitations of the study

The design of the study involved recruiting children and adolescents who showed to a greater or lesser extent the features of impulsivity we sought. Alternative approaches could contrast children with and without these features, and compare pre-, post-pubertal and young adult subjects to investigate putative developmental changes in gene expression. A comparison of groups with high comorbidity for externalizing or internalizing symptoms would be instructive both for the integrity of our concepts of impulsivity in affect and cognition, as well as for identifying the grounds for separating subgroups of ADHD with 'comorbid features'. We would also point out the severe limitations of interpreting symptoms based on lay responses to questionnaires. It is important that the preliminary work described here be extended to neuropsychological testing that would contrast not only provoked aggression with impulsive decision making, but incorporate the role of reinforcement (e.g. delayed gratification).

There are also several limitations with regard to the sample and genotyping. Although this sample is reasonably sized for moderate genetic effects, the number of individuals could be larger to detect the modest genetic effects that one would expect to observe here. In addition, individuals enrolled in the IMAGE study are from several different places throughout Europe, including Spain and Israel. Although the FBAT statistic is robust to population stratification, the PBAT screening algorithm can be affected by this. Finally, current genome-wide scans are suggesting many genes that one could not have suspected of being of interest a short time ago. Therefore some candidate genes that may now seem important were not included in this analysis.

## Conclusion

ADHD is a highly heterogeneous disorder: it has been argued that there are potentially multiple variants of the condition with different etiological bases, pathophysiological signatures and neuropsychological and behavioral markers [90]. Consequently, it has been suggested that one of the key goals for ADHD research should be to parse that heterogeneity, to create more neurobiologically homogeneous sub-groupings so as to provide more refined diagnostic criteria and more specific treatment targets [91]. One manifestation of such a strategy is the search for endophenotypes, for pathophysiologic markers that mediate the pathways from genetic and environmental risk to disorder [92]. From this perspective the current differentiation between two types of impulsiveness on the basis of their associated genes and their behavioral characteristics both highlight these constructs as putative endophenotypes for ADHD and encourage further research to explore the possibility that two 'subtypes' of ADHD might exist marked by different types of impulsiveness. The one has a strong basis in motivation and emotion with expression dependent on a degree of motor disinhibition (aggressive-behavioral impulsivity), and the other reflects poor executive control of endogenous attention-related processes that can underlie risky decision-making in several functional domains (cognitive impulsivity).

In particular, this study has taken an exploratory look at whether some SNPs from genes related to 5-HT function are associated with symptom ratings that reflect aggressive behavioral and cognitive impulsivity. Clearly different results could emerge from the study of other genes/SNPs as they become available. These SNPs cover genes for only four of the 22 types 5-HT receptor identified. There are indications that other receptors could be involved (e.g. 5-HT1D, 5-HT4, 5-HT6, 5-HT7: [71,93]. Complementary to the present study, one using cognitive impulsivity measures from neuropsychological tests (e.g. continuous performance and flanker tasks) would be necessary before one could draw firm conclusions about the influence of heritable features of the 5-HT system on cognitive decision-making processes.

Nonetheless, based on ratings of impulsive symptoms, in the first instance this study strongly implicates genetic influences in ADHD on the synthesis of adrenalin and by implication on the degree of arousal influencing the organization and expression of behavior. In the second instance we report a genetic influence on the expression of SERT, along with potential, small effects of TPH2, 5-HT1E, 5-HT2A, DAT1, DA D1 and D4 receptors prominently expressed in limbic and association cortices and implicated in the initiation of impulsive processes and their expression by young people with ADHD. These features of 3 monoamine systems and two types of impulsivity may be regarded as 'intermediate phenotypes' for the psychopathology of combined-type ADHD. The usefulness of the FBAT-PC approach in the context of dissecting impulsivity in ADHD has been demonstrated, and warrants further, closer study using measures of impulsivity defined by neuropsychological task performance.

## Abbreviations

ADHD-ct: Attention-deficit/hyperactivity disorder-combined type; BDNF: brain derived neurotrophic factor; CP/TRS: Conners parent/teacher rating scales; DA: dopamine (receptors DRD1, DRD4); DAT1 (SLC6A3): dopamine transporter; FADS2: Fatty-acid desaturase gene-2; FBAT-PC: family-based association test with principle components; IMAGE: International Multicenter ADHD Genetics study; NURR1/NR4A2: orphan nuclear receptor/transcription factor; PACS: Parental account of children's symptoms; PNMT: phenylethanolamine-N-methyl transferase; SDQ: Strengths and difficulties questionnaire; 5-HT: Serotonin (receptors 5-HT1a, b, 5-HT2a, c, 5-HT3b); SERT: serotonin transporter; SNP: single nucleotide polymorphism; STin2: Serotonin transporter [VNTR] in intron 2; TPH: tryptophan hydroxylase; VNTR: variable number tandem repeat.

## Competing interests

The authors declare that they have no competing interests.

## Authors' contributions

The first two authors (RDO, JL-S) conceived and executed the analysis, and drafted the manuscript. They were supported and advised by the working group (HC, SVF) assisted by TB, WC, and EJSS-B. Recruitment and initial data processing was performed by the CIs under the supervision and organization of the PIs who set up the IMAGE investigation (TB, JKB, RPE, MG, AM, RDO, HR, AR, JAS, HCS, PA assisted by RJLA, BF and MT) under the direction of SVF.
